# Research on the Multimodal Digital Teaching Quality Data Evaluation Model Based on Fuzzy BP Neural Network

**DOI:** 10.1155/2022/7893792

**Published:** 2022-06-11

**Authors:** Wenyan Feng, Fan Feng

**Affiliations:** ^1^School of Marxism, Dalian Ocean University, Dalian, Liaoning 116023, China; ^2^School of Marine Engineering and Technology, Sun Yat-sen University, Zhuhai, Guangdong 519000, China

## Abstract

We propose in this paper a fuzzy BP neural network model and DDAE-SVR deep neural network model to analyze multimodal digital teaching, establish a multimodal digital teaching quality data evaluation model based on a fuzzy BP neural network, and optimize the initial weights and thresholds of BP neural network by using adaptive variation genetic algorithm. Since the BP neural network is highly dependent on the initial weights and points, the improved genetic algorithm is used to optimize the initial weights and thresholds of the BP neural network, reduce the time for the BP neural network to find the importance and points that satisfy the training termination conditions, and improve the prediction accuracy and convergence speed of the neural network on the teaching quality evaluation results. The entropy value method, a data-based objectivity evaluation method, is introduced as the guidance mechanism of the BP neural network. The a priori guidance sample is obtained by the entropy method. Then, the adaptive variational genetic algorithm is used to optimize the BP neural network model to learn the a priori sample knowledge and establish the evaluation model, which reduces the subjectivity of the BP neural network learning sample. To better reflect and compare the effects of the two neural network evaluation models, BP and GA-BP, the sample data were continued to be input into the original GA and BSA to obtain the evaluation results and errors; then, the evaluation results of the two evaluation models, BP and GA-BP, were compared with the evaluation results of the two algorithms, GA and BSA. It was found that the GA-BP neural network evaluation model has higher accuracy and can be used for multimodal digital teaching quality evaluation, providing a more feasible solution.

## 1. Introduction

The mathematical model of neurons was proposed and became the pioneer of artificial neural network research [1]. Immediately after many scholars joined the study, many new theories and algorithms of artificial neural networks were proposed one after another. As a mathematical model for processing computation, the artificial neural network has been applied to problems that traditional methods and models cannot solve and achieved good results in practice. BP neural network is a multilayer feedforward network trained by an error backpropagation algorithm. The idea of the BP neural network is to correct the error signal generated in the forward propagation process using a gradient [2]. The idea of the BP neural network is to convert the error signals generated during forwarding propagation using the gradient-down method until the set accuracy target is met or the number of iterations is completed. It is then shown that any continuous function in any closed interval can be approximated by a BP network with one implicit layer. Any three-layer BP neural network can accomplish n-dimensional to m-dimensional mapping [3].

As effective feedback and adjustment tools for teaching, teaching evaluation has two definitions: macro and micro. The purpose is to optimize and improve instruction. Teaching evaluation fully implements the scientific development concept and is a milestone for education progress [4]. Teaching quality evaluation is a vital link to monitoring and improving the quality of teaching, and it is significant to conduct a systematic quality evaluation of education [5]. The development and progress of the times make the teaching methods for students in colleges and universities emerge like a spring and are full of creativity. Still, many colleges and universities' evaluation methods of teachers' teaching quality do not keep pace with the times. The ways and means are relatively single, and some colleges and universities even lack effective teaching quality evaluation models and methods. This phenomenon seriously affects the improvement of education levels in colleges and universities. In the digital society, real and virtual spaces are intertwined to form a whole new environment. While it changes the way people live and learn, it also changes the quality of their thinking and cultural characteristics [6]. Changing educational concepts and teaching methods has become a new trend in primary education. The core goal of cultivating students in the eighth essential education curriculum reform is the overall goal of the curriculum reform. As a part of primary education, junior high school IT courses should also carry out teaching activities under core literacy concepts.

Moreover, in the new junior high school IT standards, it is also clearly stated that the goal is to cultivate students' core literacy and improve their practical spirit and innovation ability [7]. To meet the requirements of the national education level and enable qualified citizens who can adapt to the digital society, IT education needs to shift from “information general knowledge” teaching to “core literacy-oriented” education. Among them, digital learning is a profound reflection of the requirements of contemporary society for human capabilities. Digital learning requires learners to make full use of digital resources, tools, and platforms to efficiently conduct independent learning and cooperative research [8]. Therefore, learners need to understand the basic operating skills and steps of using hardware and software and need to master how to study and find information online through platforms and search engines [9]. They lay the foundation for digital learning as a basic competency in information science. In addition, digital learning is also present throughout the entire IT discipline. It is not only a reliance on the other three core literacies but also a direct manifestation of them. In short, digital learning is the key to the subject of information technology, and mastering digital learning will enable you to better adapt to the development of a digital society.

## 2. Related Works

Multimodal discourse analysis theory was flourishing in the 1990s, and it involves many aspects, multimodal teaching being one of them. Multimodal teaching refers to a full range of teaching from different modalities such as visual, auditory, tactile, olfactory, and gustatory, with the help of images, text, video, audio-animation technologies, or the Internet. In its development, it must be said that the progress of technology is a crucial factor that helps it develop [10]. For example, in the beginning, the emergence of multimedia technology made teachers not only use traditional teaching tools such as the blackboard in the classroom but also use multimedia equipment, with the help of images and videos, to complete the teaching task and mobilize multiple senses to achieve the teaching goal [11]. With the progress of the times and the development of technology, communication between people has been enriched, such as telephone, video, and text chat, which are dominated by the “screen,” so nowadays, the language modality has become an alternative in communication. The role of many different modalities in touch cannot be underestimated [12]. The part of many other modalities in communication has become significant. Language teaching and learning are constantly evolving with modern technology and new ways of teaching. As more and more modalities emerge in the classroom, more and more teachers are willing to use multiple modalities in their lessons, especially in language teaching, where multimodal teaching has become a new paradigm and arguably an inevitable choice [13]. However, under the conditions of the epidemic, the offline classroom has changed to online teaching, and teachers no longer stand on the podium to teach face-to-face with students. In this situation, where the interaction between teachers and students is limited, the modalities teachers can use in the classroom are limited [14]. This paper analyzes the multimodal discourse factors and the combination of these modalities in online live teaching, analyzes the genre structure of online live classes, and concludes with research results. We hope to provide some valuable references for online teaching design and evaluation.

Digital teaching resources have received sufficient attention in Europe and the United States developed countries. The level of digital teaching has been listed as an essential aspect in the evaluation of education informatization, and “education informatization” has been listed as an international informatization development project. Digital government resource platform is a critical component of the education system; the construction and sharing of the platforms are considered the key to digital education. They are recognized by some scholars [15]. Lemon Tree School in California has centralized and established a unique online resource platform with corresponding digital teaching tools. Students can access the Internet and quickly obtain the digital resources they need. The platform built by the state of Florida, which constructed the virtual cyber school, is outstanding for its effectiveness in utilizing digital teaching resources and its exemplary effect on other platforms [16]. It is an online distance learning school specifically for elementary and middle school students, collecting many teaching resources and supplying them for free to students of all ages. At the same time, teachers communicate with students promptly through the distance education platform built by the virtual cyber school, the public social platform Facebook, and other forms to solve difficulties in the learning process and facilitate effective use of resources by students.

Most universities' current classification schemes are based on diversified criteria, mainly including essential functions, discipline types, talent cultivation levels, school scale, and management system. The classification of universities according to these criteria will produce different classification results. Liu et al. designed a research-oriented undergraduate teaching quality assurance system and extracted important factors affecting teaching quality evaluation [17]. On the other hand, Wang et al. researched the construction of a suitable teaching system in applied undergraduate institutions and provided more reliable theoretical support for cultivating applied talents and evaluating teaching quality in practice-oriented institutions through empirical research [18]. Wei et al. explored the optimization of the PBL evaluation system of medical master students and the factors influencing the teaching effect, which provided a more reliable and effective realistic basis for the research of postgraduate training and education quality evaluation [19]. Zhang et al. combined the teaching characteristics of military schools and the requirements of the Ministry of Education for military school education and established a BP neural network-based military school teaching quality evaluation system to achieve a scientific, reasonable, and timely teaching quality evaluation of military school teaching quality [20]. Yang et al. used the fuzzy comprehensive evaluation method to evaluate the teaching quality of university physics MOOC in three aspects, the degree of teaching goal achievement, the rationality of teaching content, and the appropriateness of teaching evaluation and feedback, and analyzed the problems of current university physics MOOC classroom teaching in response to the evaluation results [21]. Zhang et al. tried to use the hierarchical analysis method, which was explored and analyzed, thus providing a more scientific example basis for evaluating bilingual course teaching [22].

## 3. Fuzzy BP Neural Network Model Construction

**Figure 1:**
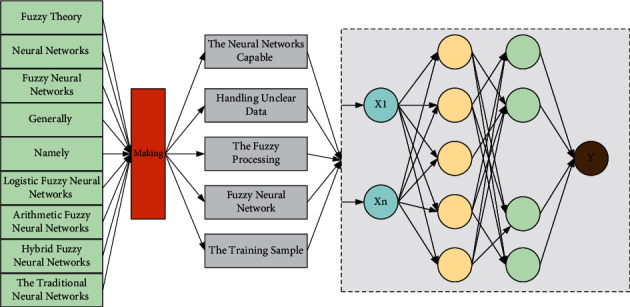
Fuzzy neural network.

At present, it is generally considered that there are three types of fuzzy neural networks, namely, logistic fuzzy neural networks, arithmetic fuzzy neural networks, and hybrid fuzzy neural networks. All three are based on the traditional neural networks with the addition of fuzzification processing, thus making the neural networks capable of handling unclear data. However, the fuzzy processing means of each of them are different. In this paper, the most used arithmetic fuzzy neural network is selected as the object of study; that is, the input information of the neural network is fuzzified by adding a fuzzification layer after the input layer, as shown in Figure 1. The input information of the neural network is the feature value extracted from the training sample, and this value is the exact value represented by the sampling point itself. These feature values can be fuzzified for initial classification to form several fuzzy sets using the affiliation function in fuzzy theory. The fuzzification process is the conversion from feature values to the affiliation degree of the sampled points.

Since BP neural networks have the disadvantage of being easily limited to local minima in the self-learning process, Mamdani fuzzy control theory is combined with BP neural networks to construct fuzzy BP neural networks, where *x* ∈ *R*^*n*^*y* ∈ *R*, let the input vector be *X*=[*X*_1_, *X*_2_, ⋯, *X*_*n*_], and make *m* corresponding affiliation functions for each input variable *x*_*i*_, where the affiliation of the variable is expressed as

(1) $$\begin{array}{c} T\left(x_{i}\right)=\frac{\sqrt{A_{i}^{1}+A_{i}^{2}}}{A_{i}^{mj}}, \end{array}$$

where *A*_*i*_^*j*^(*j*=1,2,…*m*_*i*_) is the value of the *j* linguistic variable *x*_*i*_, a fuzzy set defined on the domain *U*_*i*_, and the corresponding affiliation function is *u*_*A*_*i*__(*x*_*i*_)*i* = 1, 2,…, *n*; *j* = 1, 2,…, *m*_*j*_.

The output quantity *y* also constructs the corresponding fuzzy linguistic variables and *T*_*y*_={*B*_1_, *B*_2_,…*B*_*m*_}, where *B*^*j*^=(*j*=1,2,…*m*) is the value of *j* linguistic variables of *y*, a fuzzy set defined on the theory *U*_*y*_, and the corresponding affiliation function is *u*_*B*_*i*_^*j*^_(*y*). If a single-point fuzzy set fuzzified the input, the applicability for a given input *x* could be found for each rule as

(2) $$\begin{array}{c} a_{i}=\min\frac{uA_{2}^{j}\left(x_{2}\right)}{uA_{1}^{j}\left(x_{1}\right)}+\sqrt{uA_{n}^{j}\left(x_{n}\right)}. \end{array}$$

The affiliation function of the output fuzzy set *B* for each fuzzy rule can be obtained by fuzzy inference as

(3) $$\begin{array}{c} u_{B}\left(y\right)=\sum \frac{a_{i}}{u_{B^{i}}\left(y\right)}. \end{array}$$

Thus, the complete fuzzy set of the output quantity is *B* = ∪_*i*=1_^*m*^*B*_*i*_. If the weighted average clarification method is used, the output clarification quantity can be found as

(4) $$\begin{array}{c} y=\int \frac{yu_{B}\left(y\right)\text{d}y}{u_{B}\left(y\right)\text{d}y}, \end{array}$$

where *y*_*c*_ is the point that *u*_*B*_(*y*) takes the maximum value; it is also generally the centroid of the affiliation function

(5) $$\begin{array}{c} u_{B_{i}}\left(y\right)=\frac{\max u_{B_{i}}\left(y\right)}{a_{i}}. \end{array}$$

consisting of *n* input variables, which realize the connection with the second layer and transmit the input variables to each neuron. The second layer consists of the affiliation function, which converts the variables sent from the first layer into the corresponding affiliation pairs.

(6) $$\begin{array}{c} u_{i}=\sum_{i=1}\left(uA_{i}-x_{i}\right), \end{array}$$

where *I* = 1,2, *N*; *J* = 1,2,…, *M*_*I*_, *N* is the number of input quantities, and *m*_*i*_ the number of fuzzy partitions *x*_*i*_. For example, if the Gaussian function is used for the affiliation function,

(7) $$\begin{array}{c} u_{A}\left(x_{i}\right)=\sum \frac{\sqrt{x_{i}-a_{i}^{k}}}{\sigma_{i}^{k}}, \end{array}$$

where *a*_*i*_^*k*^ and *δ*_*i*_^*k*^ represent the center and width of the affiliation function, respectively, and the total number of nodes in this layer *n* = ∑_*i*=1_*M*_*i*_. The fourth layer has the same number of nodes as the third layer, that is, from *n*_2_ = *n*_1_ = *m*, and *w*,

(8) $$\begin{array}{c} a_{j}^{m}=\sum_{j=1}\left(\frac{a_{j}}{a_{j}-1}\right). \end{array}$$

In comparison with the clarity calculation of the standard fuzzy model given earlier, here *w*_*ij*_ is equivalent to the central value of the *j*th language-valued affiliation function. An essential property of fuzzy inference networks is their universal approximation capability, and the following theorem gives the consistent approximation performance of the fuzzy inference network system. The above equation written in vector form is then

(9) $$\begin{array}{c} w=\left\{\begin{array}{cc} w_{1}+1 & w_{2m}\\ w_{2}-1 & w_{m}-1 \end{array}\right\}. \end{array}$$

## 4. Multimodal Functions

**Figure 2:**
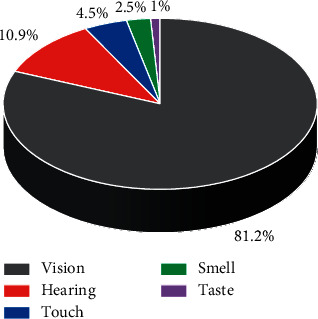
Proportion of multimodal five sensory channels to obtain external information.

The term “modality” was first introduced by the German physiologist and psychologist Helmholtz as a concept of the channels through which organisms receive information by their perceptual organs, that is, vision, hearing, touch, smell, and taste. As shown in Figure 2, the proportion of the five multimodal sensory channels for accessing external information is 81%, 11%, 4.5%, 2.5%, and 1%, in that order. On the other hand, multimodality integrates multiple senses; that is, some neuroscience and brain science scholars have shown that the amount of information received by vision is much higher than that obtained by hearing [23]. The maximum amount of information the brain receives through the eyes per second is 100 Mbps. The maximum amount of information received through the cochlea is 1 Mbps; that is, the amount of information received by vision can be 100 times that received by hearing. If images are used as information carriers, the information in the picture is five times more than that of hearing.

In multimodal classroom teaching, teachers use appropriate teaching tools and resources creatively and rationally to use multiple symbols, such as cards, wall charts, newspapers, models, objects, sketches, music, and videos, according to the teaching objectives and actual teaching environment. The traditional classroom teaching model is a teacher-centered, book-centered, and classroom-centered teaching model. These enriched teaching forms and contents realize the transformation of teaching contents from static to dynamic, from silent to audible. They can mobilize students' eyes, mouths, ears, hands, brains, and other multifunctions to help students perceive, encode, remember, store, and realize the meaning construction of knowledge. At the same time, students are permitted to build a rich experience through activities such as voiceover, speech, role-play, and presentation of English works, which allow them to move their eyes, mouths, ears, hands, and brains. The model takes advantage of the improved genetic algorithm global search and BP neural network in nonlinear mapping and reduces the influence of nonobjective factors. Only by combining the multimodal teaching theory with listening, speaking, reading, writing, and practicing, connecting the listening-teaching method, and combining various teaching methods such as listening, oral communication, situational teaching, and role-playing with individual activities, peer activities, and group cooperation can the classroom teaching be energetic, not monotonous and dull. Such a teaching mode is more in line with students' age characteristics and psychological needs, making students actively participate in classroom learning with higher interest, more concentration, longer duration, and more active thinking.

## 5. Digital Teaching Quality Data Evaluation Model Design

**Figure 3:**
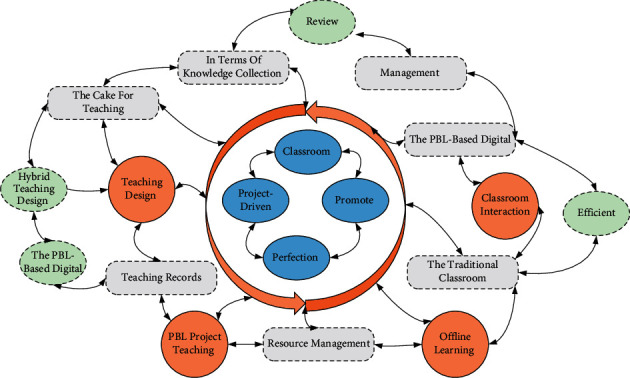
General diagram of the PBL-based digital teaching model.

The use of technology-supported digital learning tools for classroom management is one of the differences between the PBL-based digital teaching model and the traditional classroom. The proper use of a digital agency that supports classroom attendance, resource management, and teaching records can be the icing on the cake for teaching management. It can help provide a learning framework for students in terms of knowledge collection and review, classroom interaction, etc. Therefore, efficient and straightforward classroom management tools are chosen to promote the information-based instructional design toward perfection under the premise that students satisfy their own devices. According to the guiding principles of project-driven information-based teaching, the three elements of the PBL-based digital teaching model are shown in Figure 3. The participants of the PBL-based digital teaching model, hybrid teaching organizational behavior, and holographic digital teaching environment are designed. Adam's algorithm dynamically adjusts the learning step for each parameter using the gradient's first-order moment estimation and second-order moment estimation. Each iteration has a defined range of learning steps to make the parameters relatively smooth. The participants of the PBL-based digital teaching model are teachers and students, and the lecturers, assistant teachers, and expert judges are included in the teachers' team; the teaching behavior is a mixture of four types of teaching activities: PBL project teaching, classroom teaching, offline learning, and integrated classroom competition, in which project teaching is the guide and classroom teaching, offline digital learning and integrated classroom competition teaching are the realization channels of project learning; the teaching environment is the hardware environment, the hardware environment, and the hardware environment [24]. The teaching environment is the digital teaching conditions built with the support of a hardware environment, software platform, and social network as the structural support.

The theoretical research of teaching quality evaluation is how to choose the objective, accurate, and scientific assessment indexes to reflect the actual situation of teaching activities, while the technical research is a practical process, that is, to process the evaluation indexes of teaching activities and establish effective models to evaluate teaching activities affecting many factors. Therefore, the evaluation indexes should reflect the teaching process as comprehensively and objectively as possible while reducing the number of evaluation indexes as much as possible [25]. Teaching and learning are the two main subjects in teaching activities. Two evaluation indexes are determined based on students and teachers of the teaching supervision team who are most familiar with the teaching process and teaching characteristics, namely, evaluation indexes of students and evaluation indexes of teachers of the teaching supervision team in listening to lectures.

**Figure 4:**
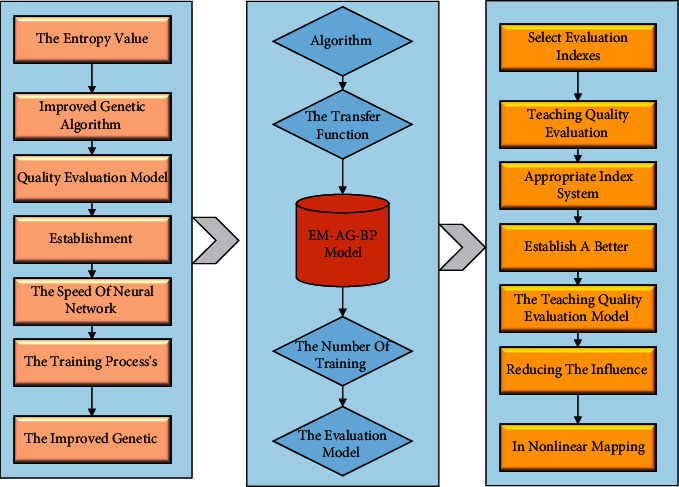
Steps of the establishing teaching quality evaluation model.

BP neural network is a neural network model with powerful nonlinear mapping ability, which can discover the linear and nonlinear patterns between data from a complex and large number of data patterns. Because of these functions of BP networks, BP neural networks are used to evaluate the quality of blended teaching, avoid the interference of human factors as much as possible, establish the evaluation model of combined education, and improve the quality assurance system of blended instruction. In the hybrid teaching evaluation of this paper, the index value of the evaluation system is used as the input value of the BP neural network, and the evaluation result is used as the output value. In the process of model evaluation of teaching quality, to make the model's evaluation and prediction performance reach the best, the relevant parameters of the model need to be set, which is currently mainly in the form of experiments. During the experimental process, the relevant parameters of the model are continuously adjusted to improve the computational power of the model and the prediction accuracy and obtain the optimal combination of the relevant parameters of the model. Then, the evaluation results of teaching quality are predicted based on the sample data. The steps to build the BP neural network model are as follows:

(1) Design of input layer: according to the established hybrid teaching evaluation system, there are 20 secondary indicators, which are used as the input vector of the neural network, so the number of neurons in the input layer is *m* = 20.
(2) Design of the output layer: in this paper, the evaluation results are used as the output of the BP network, so the number of neurons in the output layer is *n* = 1.
(3) Design of the number of layers of the hidden layer: according to the structural characteristics of the neural network and the training process and according to the Kolmogorov theory, we choose the BP network with only one implicit layer structure, and the 3-layer BP network structure can approximate any continuous function with arbitrary accuracy.
(4) Determination of the number of neurons in the hidden layer: reading the literature reveals that, in general, it is determined based on empirical formulas and multiple experiments (excellent or lousy network convergence performance), and the empirical formula chosen in this paper is as follows:
  (10) $$\begin{array}{c} l=\sum \frac{\sqrt{m-n}}{\alpha -1}. \end{array}$$
(5) Determination of neuron activation function: Considering the needs of this paper and the advantages of sigmoid function in classification and function approximation, several standard activation functions are proposed.
  (11) $$\begin{array}{c} f_{\left(x-1\right)}=\int \frac{e^{x}}{\sqrt{e^{x}-1}}. \end{array}$$
(6) Determination of model structure: according to the parameters in the above steps, the BP neural network model structure can be determined as a three-layer BP neural network of 20–7–1.
(7) Initial setting of weights and thresholds: when training modeling with BP neural networks, it is necessary to set a range of initial weights and points in the network beforehand, which is to ensure that the training does not fall on those flat areas and fall into local minima at the beginning. When setting the weights, a relatively small random number is generally taken, which can effectively shorten the learning time of the network.
This paper combines the entropy value method improved genetic algorithm [26]. The establishment process is shown in Figure 4. Adaptive variation probability is used in this genetic operation process, which enhances the speed of neural network convergence and reduces the training process's complexity. The model not only takes advantage of the improved genetic algorithm global search and BP neural network in nonlinear mapping, reducing the influence of nonobjective factors. The main modeling steps of the teaching quality evaluation model are as follows:
(1) Analyze the existing problems of teaching quality evaluation, improve them, and establish a better and more appropriate index system.
(2) Collect sample data for teaching quality evaluation and select evaluation indexes according to teachers' teaching characteristics.
(3) Determine each BP neural network algorithm parameter, including the learning rate and the number of neuron nodes in the hidden layer.
(4) The training is continuously iterated by feeding the samples into the evaluation model until the algorithm is triggered to stop.
(5) Input test samples of teaching quality evaluation to test whether the training effect of the BP neural network model optimized by the improved genetic algorithm meets the requirements and if the prediction results meet the stopping needs, go to the next step.
(6) Input the samples into the teaching quality evaluation model to derive the results.

The model takes the advantages of both the entropy method and the AGA-BP algorithm to make up for the shortcomings of the other way, which is mainly reflected in the benefit that the AGA-BP algorithm model can perform nonlinear mapping within arbitrary accuracy, which can make up for the disadvantage that the entropy method lacks the horizontal comparison of indicators [27]. The basic idea of the entropy value method, which is an objective assignment method, is to determine the indicator weights by calculating the degree of fluctuation of each indicator value; the process can reduce the deviation brought by human factors and provide a specific basis for the design of the neural network; the initial evaluation results determined by the method are used as a priori guiding samples for the AGA-BP algorithm model.

## 6. Analysis of Results

### 6.1. Analysis of the Multimodal Digital Teaching Quality Data Evaluation Model

**Figure 5:**
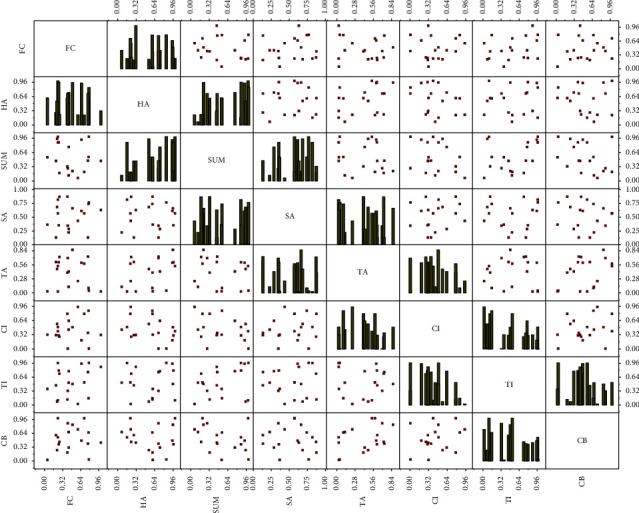
Frequency diagram of modal use in each phase of teaching with multimodal synergy.

In terms of instructional delivery stages, the modal distribution of classroom instructional stages is uneven, and the synergistic relationship between multiple modalities varies with the delivery stage. As shown in Figure 5, the ending stage (FC, HA, and SUM) has the shortest time limit, and its modal resources are relatively less distributed; the opening ceremony (CB) quickly enters the class, TI is the topic introduction, CI is the content introduction, and the modal amount is used more often than the ending stage. In contrast, the middle stage of the classroom (TA and SA) has the most extended time limit and rich activities, and the modal resources used are relatively more, with a rugby-ball type modal distribution of “big in the middle and small at the two ends,” which is in line with the standard “P–W–P” teaching mode of English reading. The primary purpose of CB (opening ceremony) is to attract students' attention and get into the class. A typical BP neural network mainly consists of an input layer, hidden layer, and output layer, where the number of neurons in each layer and the number of layers in the hidden layer need to be adjusted according to the actual situation to determine whether a reasonable network structure can reduce the number of times of network training and improve the accuracy of network learning. At the same time, TI (introduction to the topic) and CI (introduction to the content) use rich and interesting modal combinations to stimulate students' interest in learning, which takes some time. The primary purpose of FC (end of the lesson) is to say goodbye to the students and teachers and to end the class with less modal distribution and the shortest duration; meanwhile, HA (homework assignment) and SUM (summary) phases are short, with few teaching activities and little modal distribution. Secondly, the intermediate stage has more complicated teaching tasks and the most extended lecture duration.

In contrast, the intermediate stage (e.g., TA and SA) is mainly a multimodal collaboration between teachers and students through a series of activities and character interactions to construct meaning and transmit information, with the heaviest teaching tasks, significantly more modal distributions, more complex synergistic relationships, and longer duration [28]. Finally, it is influenced by the “P–W–P” model of reading. The frequency of modalities in the “reading in progress” stage was significantly higher than that in the “prereading” and “postreading” stages, reflecting a gradual and in-depth learning process.

**Figure 6:**
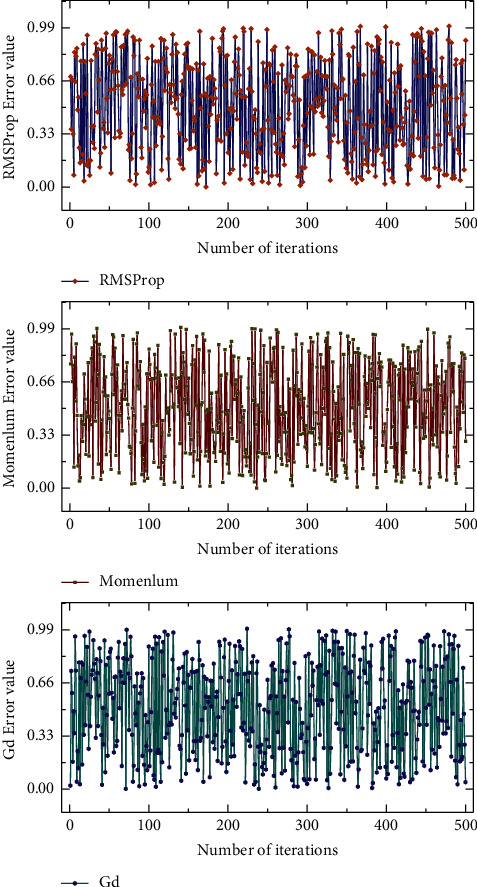
Comparison of training errors of different optimization algorithms.

Unsupervised learning training process: there are many algorithms to optimize the training process of neural network models, including the Adam algorithm. The gradient descent algorithm is currently the most popular optimization algorithm for neural network models. Still, it is slow to converge and easy to fall into local minima when approaching the minimum value. The gradient disappears during the training process of neural network models containing multiple hidden layers. Therefore, this section introduces the Adam (Adaptive Moment Estimation) algorithm as an optimization algorithm [29]. Assuming that the number of hidden layers of the DDAE-SVR deep neural network model is three and the number of neurons in the hidden layers is 20, the error between the unsupervised training output data and the original input data is calculated using the mean squared error function. The error variation curves of them are shown in Figure 6. Although the gradient descent method and the momentum algorithm have been decreasing, their decreasing speed is slow, and the number of iterations is increasing, which causes the convergence speed to be quiet. In the first 500 iterations of the RMSProp and Adam algorithms, the error between the output eigenvectors and the original data decreases rapidly; as the number of iterations increases, the error convergence tends to be flat, although the error continues to decline.

**Figure 7:**
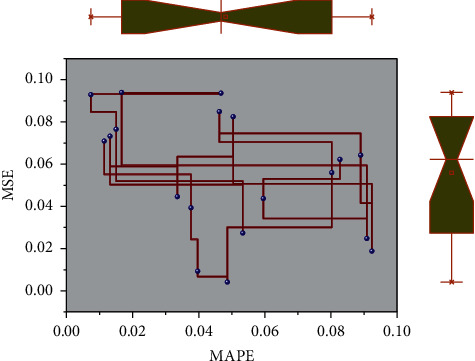
Plot of MAPE versus MSE values between predicted evaluation results and actual values.

To ensure the accuracy of the prediction evaluation of the DDAE-SVR deep neural network model, the error penalty coefficient of the support vector regression of the prediction output layer is set to 1, the kernel function is polynomial, and the parameter *v* is controlled. According to the structural characteristics of the neural network and the training process, it is known that the more the hidden layers are, the more complex the BP neural network is. According to the Kolmogorov theory, we choose the BP network with only one implicit layer structure, and the 3-layer BP network structure can approximate any continuous function with arbitrary accuracy. The system is simple and easy to implement. Then, the feature vector of the last hidden layer neuron of the model after unsupervised training is used as the input feature vector of the support vector regression, and the graphs of MAPE and MSE values between the predicted evaluation results and the actual values are shown in Figure 7. The trend of the curves of MAPE and MSE values is the same, decreasing first and then increasing with the increase of *v*. When *v* = 0.3, the model's prediction error in this chapter is the smallest, and the prediction accuracy is the best.

### 6.2. Digital Teaching Quality Data Evaluation Model Implementation

**Figure 8:**
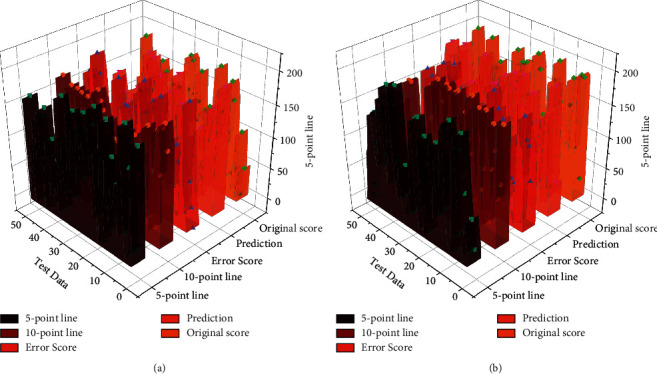
Evaluation results of GA and BSA.

In this paper, the structures of the two models were designed separately, the error analysis of the evaluation results obtained from the training was conducted, and GA-BP was established. To better reflect the effect of the two neural network evaluation models, BP and GA-BP, the original two algorithms, GA and BSA, were also used to predict the same 15 sets of test set data in the sample data for comparative analysis. The evaluation results of GA and BSA are shown in Figure 8.

Although the traditional evolutionary algorithms (GA; BSA) can produce prediction results based on each student's performance, it can be seen from the 15 sets of evaluation data that the evaluation results of GA and BSA are more variable. Even some individual evaluation results are outside the normal scoring range (100 points), while the BP neural network algorithm can predict the students' performance within the scoring range regardless of improvement. The data shows that the evaluation results of GA and BSA have more testing errors than the BP neural network evaluation model. In contrast, the predicted effects of the GA-BP neural network evaluation model are consistent with the actual results and have the slightest mistake compared with the other three; such results are more apparent when the evaluation results, as well as the errors, are made into a comparative box plot. The evaluation results of GA and BSA had more significant testing errors than the BP neural network evaluation model, while the results predicted by the GA-BP neural network evaluation model were consistent with the actual results.

**Figure 9:**
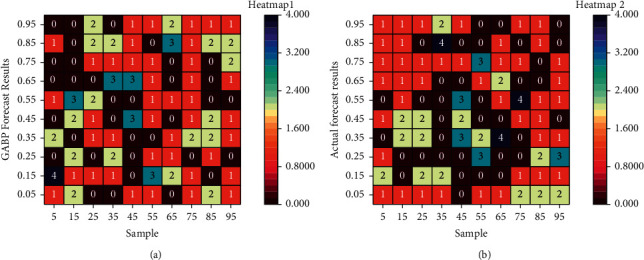
Predicted results of evaluation results.

The prediction results can be seen that the prediction error of the genetic algorithm optimized BP neural network model is significant. The predicted effects of the improved model are consistent with the actual results. As shown in Figure 9, as a comparison chart of the prediction accuracy of the three models, the enhanced model is significantly higher than the BP neural network model and the genetic algorithm optimized BP neural network model teaching quality evaluation model in terms of evaluation accuracy. Thus, the average accuracy of the evaluation of 100 sets of data based on the improved genetic algorithm BP neural network model is 84.26%. The average evaluation accuracy of the genetic algorithm optimized BP neural network model is 91. The average evaluation accuracy of the genetic algorithm optimized BP neural network model based on adaptive variation is 97.35%, which is 12.99% and 7.42% higher than the two models, respectively. The evaluation results of the BP neural network optimization method based on the adaptive variation genetic algorithm are better.

## 7. Conclusion

In this paper, we study and compare the existing basic methods of teaching quality evaluation, summarize the problems of the current teaching quality evaluation system after multiple interviews and research, and analyze the specific issues. The genetic algorithm of adaptive variation is proposed in genetic operation by examining the genetic algorithm and BP neural network's principle, structure, and characteristics. The initial threshold and weights of the BP neural network are optimized by using the improved genetic algorithm. The BP neural network model optimized by adaptive variation genetic algorithm is established. To make the neural network learning process more empirically based and avoid a priori artificial assumptions, the entropy method, which can assign weights based on the data itself and has the feature of preventing artificial subjective factors, is used as the guidance mechanism of the neural network, and a three-combined teaching quality evaluation model based on the entropy method and the adaptive genetic algorithm optimized BP neural network is established. The results show that the model can not only solve the problems of too strong subjectivity and randomness of existing evaluation methods and models, prone to overfitting and slow convergence, but also predict the optimal results of the evaluation, thus verifying the effectiveness of the model in solving the review of teaching quality in colleges and universities.

## Data Availability

The data used to support the findings of this study are available from the corresponding author upon request.
